# European Reference Networks – a flagship activity of the EU in the field of rare and complex diseases: from 2017 to 2025

**DOI:** 10.1186/s13023-026-04341-2

**Published:** 2026-04-14

**Authors:** Holm Graessner, Sophie Ripp, Alberto M. Pereira, Franz Schaefer, Irene Mathijssen, Jean-Yves Blay, Peter F. A. Mulders, Teresinha Evangelista, Marjolijn J. L. Ligtenberg, Arthur A. M. Wilde, Ruth Ladenstein, Ansgar W. Lohse, Marta Mosca, Joost Frans Swart, Francisco Hernández, Pierre Fenaux, Hélène Dollfus, Alain Verloes, Thomas Wagner, Christine Bodemer, Rene Wijnen, Maurizio Scarpa, Guillaume Jondeau, Birutė Tumienė, Sandra Gallina, Alexis Arzimanoglou, Luca Sangiorgi

**Affiliations:** 1 ERN-RND, Institute for Medical Genetics and Applied Genomics, University of Tübingen, Tübingen, Germany; 2 Centre for Rare Diseases, University Hospital Tübingen, Tübingen, Germany; 3 Institute for Medical Genetics and Applied Genomics, University of Tübingen, Tübingen, Germany; 4 Department of Endocrinology and Metabolism, Endo-ERN, Amsterdam University Medical Center, Amsterdam, The Netherlands; 5 Division of Paediatric Nephrology, Center for Pediatrics and Adolescent Medicine, ERKNet, University Hospital, Heidelberg, Germany; 6 Department of Plastic, Reconstructive and Hand Surgery, ERN CRANIO, Erasmus Medical Center, Rotterdam, The Netherlands; 7 Department of Medical Oncology, Centre Léon Bérard, Cancer Research Centre of Lyon (CRCL), EURACAN, University Claude Bernard Lyon 1, Lyon, France; 8 Department of Urology, ERN eUROGEN, Radboud University Medical Centre, Nijmegen, The Netherlands; 9 ERN EURO-NMD for Rare Neuromuscular Diseases, Responsible of the Neuromuscular Pathology Functional Unit, Neuropathology Department Hôpital Pitié-Salpêtrière, Paris, France; 10 Head Laboratory of Tumour Genetics, Department of Human Genetics, Department of Pathology, Coordinator ERN GENTURIS for Genetic Tumour Risk Syndromes, Radboud University Medical Center, Nijmegen, The Netherlands; 11 Department of Cardiology, ERN GUARD-Heart, Amsterdam UMC Location University of Amsterdam, Amsterdam Cardiovascular Science, Heart Failure and Arrhythmias, Amsterdam, The Netherlands; 12 ERN PaedCan, Children’s Cancer Research Institute - St. Anna Kinderkrebsforschung, Vienna, Austria; 13 Department of Medicine, ERN RARE-LIVER, University Medical Centre Hamburg-Eppendorf, Hamburg, Germany; 14 Rheumatology Unit, ERN ReCONNET, Azienda Ospedaliero Universitaria Pisana and Department of Clinical and Experimental Medicine, University of Pisa, Pisa, Italy; 15 ERN-RITA for Rare Immunological Disorders, Pediatric Rheumatology and Immunology Department, Wilhelmina Children’s Hospital / UMC Utrecht, Utrecht, The Netherlands; 16 ERN TransplantChild, Paediatric Surgery Department, La Paz University Hospital, Congenital Malformations and Transplantation Group, La Paz Institute for Health Research (IdiPAZ), Madrid, Spain; 17 Département d’hématologie et immunologie (DMU), ERN-EuroBloodNet, APHP Nord, Service d’hématologie séniors, Hôpital St Louis, Université Paris Cité, Paris, France; 18 ERN-EYE, Centre de Référence Pour les Affections Rares en Génétique Ophtalmologique (CRMR CARGO), Institut de Génétique Médicale d’Alsace (IGMA), FSMR SENSGENE, Hôpitaux Universitaires de Strasbourg, France, Université de Strasbourg, UMRS_1112, Strasbourg, France; 19 Department of Genetics, ERN-ITHACA, AP-HP - Université de Paris, INSERM UMR 1141 “NeuroDiderot”, Hôpital Robert Debré, Paris, France; 20 ERN-LUNG, Frankfurt Reference Center for Rare Diseases (FRZSE), Universitätsklinikum Frankfurt am Main, Frankfurt am Main, Germany; 21 Department of Dermatology Necker Enfants Malades Hospital, ERN-Skin, APHP, Paris Cité University, Paris, France; 22 Department of Pediatric Surgery, ERNICA, Erasmus MC Sophia Children’s Hospital, Rotterdam, Netherlands; 23 Regional Coordinating Center for Rare Diseases, MetabERN, Udine University Hospital, Udine, Italy; 24 ERN VASCERN for Rare Multisystemic Vascular Diseases, Centre de référence pour le Syndrome de Marfan et Apparentés, Department of Cardiology, AP-HP, Université Paris Cité, Hôpital Bichat-Claude Bernard, INSERM U1148, Hopital Bichat Paris, VASCERN HTAD European Reference Centre, Paris, France; 25 Faculty of Medicine, Vilnius University, Institute of Biomedical Sciences, Vilnius, Lithuania; 26 Health and Food Safety, European Commission, Bruxelles, Belgium; 27 ERN EpiCARE for Rare and Complex Epilepsies, Epilepsy Unit, Neurology Department Hospital Sant Joan de Déu, Barcelona, Spain; 28 Department of Rare Skeletal Disorders, ERN BOND, IRCCS Istituto Ortopedico Rizzoli, Bologna, Italy

**Keywords:** European Reference Networks, Rare diseases, Cross-border healthcare, European health union, Clinical patient management system, JARDIN, Patient-centred care, EU4Health

## Abstract

**Background:**

Although individual rare and complex diseases (RDs) affect small patient populations, together they impact an estimated 27–36 million people across the European Union. Addressing this major public health challenge has been a long-term priority for the European Union, leading to the establishment of the European Reference Networks (ERNs) in 2017.

**Main body:**

ERNs are cross-border networks connecting clinical expert centres to share knowledge, improve and harmonise diagnosis and care for patients with rare and complex diseases. Since their inception, 24 ERNs have united 1,606 expert centres across 375 hospitals in all EU Member States and Norway. Their activities span multidisciplinary clinical collaboration, patient-centred governance, education and training, and the development of clinical guidelines. Over 4900 extremely rare or difficult cases have been discussed among experts without requiring the patients to travel abroad when expertise was not available in their own countries. A key factor for this success is the cross-border IT platform - known as the Clinical Patient Management System 2.0 - provided by the European Commission for medical discussions, which enables experts to share patient data, including medical images and lab results, in a secure and protected environment that is fully compliant with all relevant security and data privacy requirements. ERNs have demonstrated resilience in crises such as the COVID-19 pandemic and the war in Ukraine, providing rapid, coordinated responses to sustain care for vulnerable patient groups. The first formal evaluation in 2023 confirmed that more than 95% of member centres met quality standards, underscoring the networks’ maturity and effectiveness. Moving into the next phase, the Joint Action JARDIN (2024–2027) aims to integrate ERNs into national healthcare systems to ensure sustainability and equitable access to high-quality RD care.

**Conclusions:**

ERNs exemplify European solidarity and innovation in healthcare, transforming how rare disease expertise is shared and applied across borders. Their continued integration into national systems will be pivotal to achieving a truly cohesive European Health Union that delivers improved outcomes for all patients with rare and complex diseases.

Whilst rare and complex diseases (RD) are uncommon, the number of citizens affected by them are not: in the EU alone, estimations are that between 27 and 36 million people live with a rare disease. Significant resources have therefore been deployed over time by the European Commission to address this key public health issue and support patients and families affected by a rare disease.

The European Commission has been active in addressing rare diseases for more than 30 years, first identified as a public health priority in the Commission in November 1993.

EU policy and action in the field of rare diseases is legally underpinned and guided by:

- The Commission Communication on ‘Rare diseases: Europe’s challenges’^1^,
- The Council Recommendation on an action in the field of rare diseases^2^,
- The Directive 2011/24/EU on patients’ rights in cross-border healthcare^3^.

One of the true European success stories that comes from this foundation are the 24 European Reference Networks (ERNs). These are cross-border networks that bring together centres of expertise and reference in different European hospitals to help patients. ERNs enable specialists in Europe to share expertise on cases of patients affected by rare, low-prevalence and complex diseases, providing advice on the most appropriate diagnosis and the best treatment available.

Together, these networks leverage the collective expertise for patient’s care and are a prime example of European solidarity and innovation, allowing the expertise to travel, rather than patients.

The added value of the ERNs’ daily work demonstrates the critical need for multidisciplinary care in rare and complex diseases. Within and across networks, ERNs facilitate collaboration across medical disciplines to ensure comprehensive patient-centred care. Joint sessions at scientific congresses are regularly organised to discuss advances in rare disease research and care. In parallel, ERN project management teams, funded by the EU, work collectively to disseminate information across defined disease areas, reinforcing collaboration, sharing resources, and enhancing training to improve patient care throughout Europe. All 24 ERNs are also working closely with disease-specific patient advocacy groups and associations to ensure that patients’ perspectives remain at the centre of the activities.

Thanks to EU4Health programme^4^ funding amounting to 77.4 million euro, the ERNs, at present, bring together 1.605 expert centres working in 375 hospitals located across the 27 Member States of the European Union and Norway. As such, ERNs have raised the level of collaboration and interaction to an unprecedented level, both in terms of quantity and quality.

In 2023, 24 European Reference Networks, including 836 members, completed their first evaluation^5^. Overall, the evaluation concluded that the ERN ecosystem is functioning well, providing highly specialist deliverables for rare disease patients such as consultations for diagnosis and therapies, the production of clinical guidelines and specialised trainings.

100% of the ERN networks and 88% of their members obtained satisfactory results, while only 4% of the members saw their membership terminated and 8% (72 Clinical Centres) were asked to submit an improvement plan. After the re-evaluation process in 2025, a total of 799 centres out of 836 evaluated clinical centres successfully passed the first periodic evaluation, which is about 95.6%. The membership of 37 (4.4%) clinical centres was terminated. Ultimately, the evaluation confirms the Networks have delivered on many areas of specialist work since their establishment. Since 2017, when the ERNs were officially established, the ERN system can be broadly divided into three phases.

Phase one was a process of *learning-by-doing*, which – among others - involved the ERNs, the European Commission, the EU Member States, patient organisations, hospitals and clinicians. During this time, the overall ERN system began to take shape and develop, creating the innovative EU structure that we have today.

After this initial phase, the ERN system evolved into phase two: *consolidation*. The focus shifted towards strengthening inter-ERN coordination, ensuring homogeneity of core activities and integrating key performance indicators to help showcase the impact of the Networks’ activities.

The system now stands at the beginning of phase three, which will address the primary challenge of achieving the full potential of the European Reference Networks: *integration of the ERNs into the national healthcare systems.* To this end, the Commission launched the Joint Action JARDIN to integrate the ERNs into national healthcare systems and help shape Union policies and programmes, and support Member States in the elaboration of national strategies. This should also facilitate the integration of the ERNs in the national healthcare systems to improve the care, diagnosis and treatment of rare disease patients and families. The Joint Action has a budget of 18.75 million Euros, covering a period of three years 2024–2027 and involves all EU Member States, Norway, and Ukraine as well as all key stakeholders involved in this field, such as patients’ organisations and hospital managers.

To highlight the need for sustainability and integration of the ERNs’ work, expertise and impact, this article provides an overview of the infrastructures the ERNs have been establishing and the different activities and initiatives they have been pursuing.

## Healthcare and clinical patient management system

In 2017, the first year of the ERNs’ existence, the centres participating in the ERNs indicated that they supported 161,379 patients with the diagnosis of a disease or condition within their medical scope. By 2024, this number increased to 416,234 patients, marking a growth of 160% over the course of 6 years of activities.

Alongside facilitating patient referrals, the Commission has financed the creation of the Clinical Patient Management System (CPMS). This tool is a secure and dedicated IT platform, that facilitates the work of the ERNs to provide cross-border clinical expertise by supporting cross-border medical discussions among healthcare professionals. In 2025, the Commission updated the tool to version 2.0 which enables a customised, more flexible, secure and user-friendly environment for healthcare professionals to share patient information, discuss cases and develop joint treatment plans. It also improves the quality and timeliness of patient care, supports medical imaging, improved data quality and data analysis whilst being fully compliant with the General Data Protection Regulation. The platform is also accessible via a dedicated portable application. As of February 2025, all ERNs are now using the new version of the IT platform.

Over the first seven years of their existence, the Networks’ experts have discussed and provided multidisciplinary EU cross-border medical discussions on diagnosis, treatment and disease management for more than 4.900 extremely rare or difficult cases via the Clinical Patient Management System. This has allowed expertise, rather than people, to travel across borders for the delivery of high-quality care in respective care pathways [1–14]. CPMS uptake currently varies across ERNs. Structural clinical use-case-based implementation has been achieved by some ERNs with diagnosis, therapy decision and rare case constellation being key use cases. Tangible clinical decision benefits are reported. Key barriers include technical complexity, time/workflow burden, national data-protection requirements, and different training/helpdesk support.

The Clinical Patient Management System also allows early career experts to gain invaluable experience by participating in case discussions led by senior experts, learning how to prioritise investigations and treatments to achieve optimal patient outcomes.

With successful tools in place, the system for cross-border medical discussions among experts can now be leveraged to its full extent.

## Patient centeredness

The ERNs put patient needs at the centre of their activities [15–66]. Patient involvement in ERNs takes place through the 24 European Patient Advocacy Groups (ePAGs), aligned to each ERN, with advocates formally embedded in ERN boards/working groups to ensure patient priorities inform governance, outputs and evaluation activities. Empirical work shows how this translates into practice: patient advocates participating in working groups on the development of guidelines, usage of large multi-country patient surveys to inform about education and quality improvement [56] as well as co-design and co-production of patient journeys^6^. However, implementation is variable across ERNs, with challenges including workload/time constraints for patient representatives, language, and uneven support structures for participation. Furthermore, there is a strong collaboration with EURORDIS – a non-profit alliance of over 1000 rare disease patient organisations from 74 countries – alongside a large number of patient organisations.

Patient advocacy organisations also act as educational resources. Patient advocates can learn more about disease diagnosis, treatment, and research methodologies. The presence of these organisations ensures that patients’ perspectives are not only listened to, but integrated into the clinical, organisational and strategic decision-making processes of ERNs. Patient representatives actively contribute to the definition of diagnostic and therapeutic pathways, the validation of educational tools and the construction of models of cross-border care. Their contribution is even more relevant because it is anchored to the direct experience of life with the disease, and therefore capable of directing health choices towards a real centrality of the person.

For example, nearly 150 scientifically validated information leaflets and Patient Journeys were developed across the ERNs in collaboration with patient associations, providing an invaluable knowledge resource for patients and family doctors. The ePAG led co-production of Patient Journey leaflets, which are info-graphical overviews of rare disease patients’ needs, and which are available in all EU languages, is a key achievement of ERNs. All such scientifically validated documentation can be freely downloaded from the ERNs’ websites and used by physicians and caregivers.

## Training and education

Rare and complex diseases present significant challenges in the education and training of healthcare professionals due to their low prevalence, limited evidence-based knowledge, and the rapid evolution of diagnostic tools, techniques, and treatment discoveries. Since their establishment in 2017, the 24 ERNs have worked on: (i) identifying and addressing training gaps and requirements; (ii) encouraging and facilitating the development of training and continuous education programs for early career experts and other healthcare providers within and beyond the Networks; (iii) organizing teaching and training activities, including exchanges; and (iv) developing and contributing to patient education strategies [67–88].

Leading clinicians, scientists, and healthcare providers within ERNs contributed their expertise to producing scientifically validated training and education resources for healthcare professionals. To disseminate knowledge, all ERNs developed open-access webinars presented by leading European experts. In the years 2020–2024, these activities have surged, resulting in more than 1,030 educational webinars, many incorporating multidisciplinary presentations. These webinars, now widely utilized in general hospital teaching sessions, exemplify state-of-the-art education for healthcare providers. In addition, more than 540 face-to-face educational activities, including workshops, seminars, and seasonal schools, were organized during this period. The European Commission also funded pilot exchange programs enabling short-term immersions in accredited clinical teams, fostering knowledge exchange, skills development, and cross-border collaboration. Over 350 individuals participated in these programs, tailored to the specificities of each ERN.

Structuring training activities in a sustainable fashion, ERNs have been establishing postgraduate training programs to educate the new generation of experts, thereby reflecting European training requirements that are adopted and approved by the European Union of Medical Specialties and respectively appraised examinations.

A robust network of trust and collaboration among clinical teams, underpinned by shared scientific practices, is now in place to provide cutting edge training and education activities across Europe.

ERNs increasingly link training “reach” metrics (participants, countries, materials) to effectiveness measures such as learning analytics and post-activity evaluations. Sustainability is supported by re-usable digital formats and will be facilitated by establishing digital databases and stronger integration into national training structures.

## Clinical patient guidelines and other clinical decision support tools

In the years 2020–2024, the ERNs have also endorsed or developed more than 530 guidelines and recommendations, tailored to specific rare diseases to standardise care and enhance education across Member States. These guidelines significantly contribute to improving clinical practices [12, 89–337]. Additionally, ERNs provided evidence to policymakers to advocate for equitable access to diagnostic tools, such as genetic screening, and treatments [16, 145, 338–374].

## ERNs in emergency situations

During their 7 years of existence, ERNs faced two major crises: the COVID-19 pandemic and Russia’s war of aggression against Ukraine. In these extraordinary scenarios, the 24 Networks and their members concentrated their effort in setting up and participating in initiatives aimed at supporting both patients and their caregivers and healthcare professionals dealing with rare diseases [375–415]. Generally, ERNs promptly reacted by creating a COVID-19 emergency webpage in their websites and tailored their communication strategy to immediately become a hub for the rare disease community.

In the case of the Russia’s war of aggression against Ukraine, the ERN coordinators spontaneously decided to initiate a concerted action to support patients from Ukraine: already in March 2022, they deployed an almost instant response via a devoted website [erncare4ua.com] to help Ukrainian patients contact the 24 coordinating ERN teams liaising with their members and relevant patient organisations for specialised medical support. In addition to remote support, hundreds of cases have been referred to ERN medical teams which successfully organised multidisciplinary visits for affected Ukrainian refugees and provided support to healthcare professionals for the treatment of their patients located in Ukraine. Similarly to the COVID-19 emergency, all ERNs published a dedicated webpage on their websites focusing on assistance in their area of expertise and produced tailored educational products for healthcare professionals and patient advocates. A webinar series has been produced with simultaneous translation for Ukrainian patients and clinicians, including contributions from Ukrainian doctors.

This crisis-response experience highlights ERNs as a resilience asset. Key lessons are the importance of “digital-first” coordination, pre-defined escalation pathways, and better linkage between ERNs and national emergency/referral systems to reduce variability in access during shocks.

Looking forward, via the Joint Action for the Integration of ERNs into national healthcare systems (jardin-ern.eu), ERNs are now proposing to draft a suggested process for identifying crisis or emergency situations that may require a concrete and harmonised response for the benefit of the whole rare disease community.

## Registries

Additional core activities, that lie within the healthcare scope of the ERNs, are the set-up of patient registries that contain interoperable patients’ data seen by the ERNs. Registries allow patients’ data to be harmonised via defined classification and ontologies (e.g., Orphanet for the disease classification or disease-specific ontologies) to enable the identification of patient cohorts for clinical research across different countries, identification of statistically significant cohorts for rare diseases and the generation of epidemiological information [416–462]. Furthermore, the registries function as a crucial platform linking care to research.

ERN registries apply GDPR-compliant data governance and include data access processes for secondary use. ERN registries align with the EU RD Platform (JRC)^7^ building blocks, including the Set of Common Data Elements (EU-CDEs) and ERDRI tools (e.g., ERDRI.mdr metadata repository to support semantic interoperability). Links to broader European research infrastructures are being strengthened, for example with the ERN registry FAIRification [463] and interoperability support embedded in initiatives such as the European Joint Programming for RD [464] and the European Rare Disease Research Alliance (ERDERA).

ERNs also act as a key structure to allow the collection of large enough populations of patients with rare diseases through international European collaboration (between others SOLVE-RD [solve-rd.eu]; ERICA [erica-rd.eu]; ERDERA [erdera.org]; RealiseD [realised-ihi.eu]) to facilitate clinical research and trials.

In ERNs, cross-border *clinical* data exchange is primarily handled through the GDPR-aligned CPMS IT platform for medical discussions; FAIRification and (in some networks) federated querying are being developed mainly in the *registry/research* layer [430], but these are not yet systematically implemented across all ERNs, and federated learning remains an emerging rather than standardised ERN-wide approach.

## Conclusion

The health impact of ERNs can be comparable to ensuring life-saving drug accessibility and foster sustainability, capacity building and empowerment on the national level. They also help to address critical healthcare issues such as availability, accessibility and quality of diagnostic practices including newborn screening.

Furthermore, ERN impact is increasingly approached through outcome-oriented measurement aligned to diagnostic pathways, clinical practice and cross-border access to expertise. A key enabler is the annual ERN continuous monitoring framework^8^, which collects harmonised indicators across all ERNs, supporting longitudinal comparisons across networks and Member States^9^. At network level, concrete outcome signals are increasingly emerging linked to specific ERN activities such as CPMS based use cases, ERN registry data collection and analysis [416], as well as implementation of healthcare quality cycles [465]. Also, cross-ERN registry harmonisation efforts are beginning to operationalise pathway outcomes such as time to diagnosis, utilising interoperable data elements of all ERN registries. Today, the ERNs represent well-structured and highly interconnected networks that have built strong collaborative relationships with the respective European professional societies. Although still in the consolidation phase, ERNs are already having a significant impact on how care is being provided to patients with rare diseases in Europe. The true potential of ERNs, however, will only be reached when the ERNs are more efficiently linked to the national health systems. That is the aim of the three-year Joint Action JARDIN.

Ultimately, ERNs epitomise the concrete added value and successful results that can come from strong European collaboration and networking in health. ERNs lie at the heart of a strong European Health Union, which aims to improve the health of all citizens, no matter where they live, no matter their disease or complex condition. The accumulated experience and the cross-border dimension of the ERNs place them as a flagship initiative in the European Health Union.

**Addendum 1:** Websites of all 24 European Reference Networks

Acronym	Name	Website
Endo-ERN	European Reference Network on rare endocrine conditions	https://endo-ern.eu/
ERKNet	European Reference Network on rare kidney diseases	https://www.erknet.org/
ERN BOND	European Reference Network on rare bone disorders	https://ernbond.eu/
ERN CRANIO	European Reference Network on rare craniofacial anomalies and ear, nose and throat (ENT) disorders	https://www.ern-cranio.eu/
ERN EpiCARE	European Reference Network on rare and complex epilepsies	https://epi-care.eu/
ERN EURACAN	European Reference Network on rare adult solid cancers	https://www.euracan.eu/
ERN eUROGEN	European Reference Network on rare uro-recto-genital diseases and complex conditions	https://eurogen-ern.eu/
ERN EURO-NMD	European Reference Network on rare neuromuscular diseases	https://ern-euro-nmd.eu/
ERN GENTURIS	European Reference Network on rare genetic tumour risk syndromes	https://www.genturis.eu/
ERN GUARD-Heart	European Reference Network on uncommon and rare diseases of the heart	https://guardheart.ern-net.eu/
ERN PaedCan	European Reference Network on paediatric cancer (haemato-oncology)	https://paedcan.ern-net.eu/
ERN RARE-LIVER	European Reference Network on rare hepatological diseases	https://rare-liver.eu/
ERN ReCONNET	European Reference Network on rare connective tissue and musculoskeletal diseases	https://reconnet.ern-net.eu/
ERN RITA	European Reference Network on rare immunodeficiency, autoinflammatory, autoimmune, and paediatric rheumatic diseases	https://ern-rita.org/
ERN TRANSPLANT-CHILD	European Reference Network on transplantation in children	https://transplantchild.eu/
ERN-EuroBloodNet	European Reference Network on rare haematological diseases	https://eurobloodnet.eu/
ERN-EYE	European Reference Network on rare eye diseases	https://www.ern-eye.eu/
ERN-ITHACA	European Reference Network on rare malformation syndromes, intellectual and other neurodevelopmental disorders	https://ern-ithaca.eu/
ERN-LUNG	European Reference Network on rare respiratory diseases	https://ern-lung.eu/
ERN-RND	European Reference Network on rare neurological diseases	https://www.ern-rnd.eu/
ERN-Skin	European Reference Network on rare, complex, and undiagnosed skin disorders	https://ern-skin.eu/
ERNICA	European Reference Network on rare inherited and congenital (digestive and gastrointestinal) anomalies	https://www.ern-ernica.eu/
MetabERN	European Reference Network on hereditary metabolic disorders	https://metab.ern-net.eu/
VASCERN	European Reference Network on rare multisystemic vascular diseases	https://vascern.eu/

## Data Availability

Not applicable.

## Abbreviations

COVID-19: Coronavirus Disease
CPMS: Clinical Patient Management System
ePAG: European Patient Advocacy Group
ERN: European Reference Network
EU: European Union
JARDIN: Joint Action for the Integration of ERNs into national healthcare systems
RD: Rare and Complex Diseases

## Footnotes

1 Communication from the Commission to the European Parliament, the Council, the European Economic and Social Committee and the Committee of the Regions on Rare Diseases - Europe’s challenges.

2 Council Recommendation of 8 June 2009 on an action in the field of rare diseases.

3 Directive 2011/24/EU of the European Parliament and of the Council of 9 March 2011 on the application of patients’ rights in cross-border healthcare.

4 EU4Health programme 2021–2027 – a vision for a healthier European Union - European Commission.

5 Commission Implementing Decision 2014/287/EU of 10 March 2014 ‘setting out criteria for establishing and evaluating European Reference Networks and their Members and for facilitating the exchange of information and expertise on establishing and evaluating such Networks’, as amended by the Commission Implementing Decision (EU) 2019/1269 of 26 July 2019, and in particular Article 12 and Article 14 thereof.

6
https://www.ern-rnd.eu/disease-knowledge-hub/ern-rnd-patient-journeys/.

7
https://eu-rd-platform.jrc.ec.europa.eu.

8 ERN Continuous Monitoring and Quality Improvement System - Public Health.

9
https://health.ec.europa.eu/publications/european-reference-networks-monitoring-report-2025_en.
